# New insights into the adaptive transcriptional response to nitrogen starvation in *Escherichia coli*

**DOI:** 10.1042/BST20180502

**Published:** 2018-12-04

**Authors:** Amy Switzer, Daniel R. Brown, Sivaramesh Wigneshweraraj

**Affiliations:** MRC Centre for Molecular Bacteriology and Infection, Imperial College London, London SW7 2AZ, U.K.

**Keywords:** *Escherichia coli*, nitrogen metabolism, RNA polymerase, transcriptomics

## Abstract

Bacterial adaptive responses to biotic and abiotic stresses often involve large-scale reprogramming of the transcriptome. Since nitrogen is an essential component of the bacterial cell, the transcriptional basis of the adaptive response to nitrogen starvation has been well studied. The adaptive response to N starvation in *Escherichia coli* is primarily a ‘scavenging response’, which results in the transcription of genes required for the transport and catabolism of nitrogenous compounds. However, recent genome-scale studies have begun to uncover and expand some of the intricate regulatory complexities that underpin the adaptive transcriptional response to nitrogen starvation in *E. coli*. The purpose of this review is to highlight some of these new developments.

## Introduction

Conditions that sustain constant bacterial growth are seldom found in nature. Bacterial growth is often limited by the availability of nutrients: soil, water, and even host environments such as macrophages can lack essential nutrients to support growth. Hence, many bacteria spend the majority of their time in states of little or no growth because they are starved of essential nutrients. The nutrient-starved and growth-attenuated state is widely considered an important physiological condition in bacterial pathogenesis and survival and is thus an area of intense research. Nitrogen (N) is an essential element of most macromolecules in the bacterial cell (including proteins, nucleic acids, and cell wall components). Many bacterial pathogens experience nitrogen limitation in host environments such as the urinary tract (e.g. uropathogenic *Escherichia coli* [1]) or macrophages (e.g. *Salmonella* Typhimurium [2]) and respond by activating specific adaptive processes. Emerging evidence also indicates that bacterial nitrogen metabolism and nitrogen stress responses are important for the gut microbiota to be properly established and maintained [3]. Bacteria can assimilate a variety of N sources where ammonia typically supports the fastest growth, serving as the preferred N source for many bacteria including *E. coli* [4]. The adaptive responses that allow bacteria to cope with, and survive, N starvation primarily manifest themselves through large-scale changes in the transcriptome. This review summarises the transcriptional basis underpinning how *E. coli* adapts to N starvation, specifically highlighting two new branches in this important adaptive process.

## A sensing mechanism for N starvation

**Figure 1.**
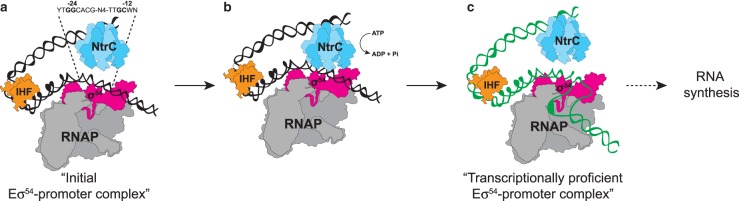
Steps involved in transcription initiation by the major variant bacterial RNAP, Eσ^54^, which is required for the expression of the NtrC regulon in N-starved *E. coli*. In (**a**), Eσ^54^ forms the initial promoter complex, which is transcriptionally silent. The enhancer DNA-bound NtrC interacts with the initial Eσ^54^ promoter complex by looping out the intervening DNA in an ATP hydrolysis-dependent manner (**b**), leading to the formation of the transcriptionally proficient Eσ^54^ promoter complex (**c**).

**Figure 2.**
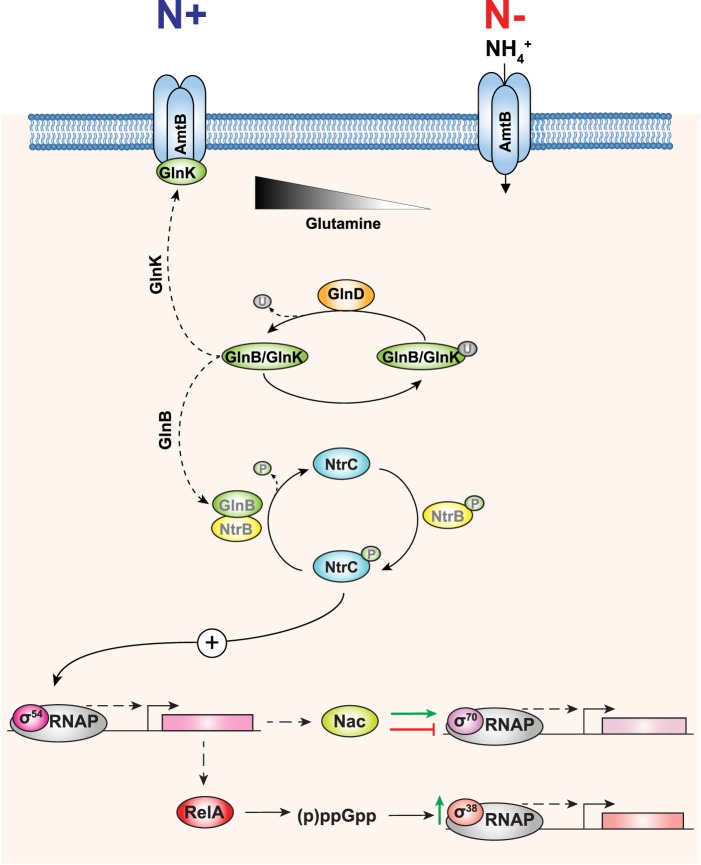
The Ntr response in *E. coli*. The signalling cascade *E. coli* uses to sense and respond to N starvation, which results in reprogramming of the transcriptome by least three different forms of RNAP.

The initial transcriptional response to nitrogen starvation is dependent on the RNA polymerase (RNAP; E) containing the major variant promoter-specificity sigma (σ) factor subunit σ^54^ (reviewed in ref. [5]). In contrast with the major *E. coli* σ^70^ family of σ factors involved in adaptive responses to diverse stress cues, including housekeeping functions, σ^54^ directs its RNAP (Eσ^54^) to promoters uniquely characterised by consensus dinucleotide sequences positioned at −24 and −12 relative to the transcription start site at +1. Promoter complexes formed by Eσ^54^ initially exist in a transcriptionally inactive state until activated by a specialised activator protein, which binds to enhancer-like sequences positioned ∼100–150 base pairs upstream of the +1 site and interact with the Eσ^54^ at the promoter by looping (assisted by the DNA-bending protein integration host factor) out the intervening DNA. The transcriptionally proficient promoter complex is only formed when the activator protein remodels the initial promoter complex in a reaction consuming ATP (Figure 1) [6]. In Enterobacteria, the master transcription regulator of the adaptive response to N starvation, the Nitrogen regulation (Ntr) stress response, is NtrC of the NtrBC two-component system, where NtrB is the cognate histidine kinase of NtrC. Under N replete growth, ammonia is converted to glutamine and glutamate, which are the primary nitrogen donors in the cell. The intracellular concentration of glutamine is thought to be the main intracellular signal for N availability in *E. coli* and its levels are detected by the uridylyltransferase/uridylyl-removing enzyme, GlnD (Figure 2). We refer readers to reviews by Dixon and Kahn [7], Larry Reitzer [4] and Huergo et al. [8] for a detailed description of the mechanism by which glutamine is sensed by UT/UR (uridylyltransferase/uridylyl-removing). At low glutamine concentrations, GlnD deuridylylates the PII homologues, GlnB and GlnK. The deuridylylated form of GlnB binds to NtrB to activate its phosphatase activity and consequently dephosphorylates NtrC. The deuridylylated form of GlnK interacts with the ammonium transporter, AmtB, to inhibit ammonium uptake. Conversely, under N sufficiency, when the intracellular concentration of glutamine is high, GlnB and GlnK become deuridylylated, which results in (i) the phosphorylation of NtrC (by NtrB) leading to expression of the NtrC regulon and (ii) the inhibition of the interaction of GlnK with AmtB, thus enabling the uptake of ammonium (Figure 2).

## The expanded regulon of NtrC

Previous studies from Sydney Kustu and colleagues dubbed the adaptive response to N starvation as a scavenging response because many of the genes that are activated by NtrC encode transport systems for nitrogenous compounds [9]. One of the NtrC-activated genes encodes the N assimilation control (Nac) protein, which is a DNA-binding dual transcription regulator. Nac serves to both activate and repress Eσ^70^-dependent genes required for adaptation to N starvation. Nac-regulated genes are involved in diverse physiological processes ([10], and our unpublished data). We refer readers interested in the structure–function–regulation of Nac to a detailed review by Bob Bender [11]. Recently, a study by Brown et al. [12] highlighted the need for *E. coli* to integrate the specific adaptive response to N starvation with bacterial stringent response-mediated changes in gene expression to allow the cell to optimally cope with low N availability. By combining global gene expression analysis with genome-wide profiling of NtrC-binding sites in N-starved *E. coli*, Brown et al. discovered that NtrC also activates the transcription of *relA*; the product of which is responsible for the synthesis of the major stress signalling nucleotide, (p)ppGpp [13,14]. Accumulation of (p)ppGpp has far-reaching consequences for major cellular processes, including transcription, translation and DNA replication, which collectively form the cell's stringent response. The stringent response leads to down-regulation of stable RNA synthesis (rRNA and tRNA), required in high abundance for fast-growing cells, while biosynthetic operons are up-regulated to promote survival until growth conditions improve. (p)ppGpp also induces intracellular accumulation of σ^38^ (the predominant σ factor active in stationary phase *E. coli*) and thus transcription from Eσ^38^-dependent promoters [15]. In essence, in its simplest form, the adaptive transcriptional response to N starvation, initiated by the transcription regulators, NtrC and Nac, can be considered to represent the integration of at least three distinct regulons (however, see later), which are dependent on the three different forms of the bacterial RNAP, namely Eσ^54^, Eσ^70^ and Eσ^38^ (Figure 2). Consequently, the *initial* transcriptional response to N starvation results in the global reprogramming of transcription, involving ∼40% of all *E. coli* genes, to allow *E. coli* cope with N starvation.

## A role for a kinase in the adaptive transcriptional response to N starvation

Until recently, the roles of all but one — the *yeaGH* operon — of the transcription units within the NtrC regulon during adaptation to N starvation were well understood. The transcription of the *yeaGH* operon is driven by two adjacently positioned promoters, which are dependent on Eσ^54^ and Eσ^38^, respectively. Thus, unsurprisingly, *yeaGH* is one of the most highly expressed operons during the initial adaptive transcriptional response to N starvation [9,12]. Intriguingly, the *yeaGH* operon is also highly expressed in *E. coli* and *Salmonella* in response to diverse stresses including low pH [16], hyperosmotic conditions [17], entry into stationary phase [16], nitrogen starvation [12], sulfur limitation [18], in biofilms [19] and exposure to antimicrobial peptide Polymyxin B [20], clearly suggesting that the products of *yeaG* and *yeaH* have a role in how bacteria adapt to these stresses. The *yeaGH* operon is highly conserved across several bacterial species, particularly Enterobacteria, where both genes are present in numerous pathogenic species, including *Salmonella*, *Shigella*, *Yersinia* and *Klebsiella*, while *prkC*, the homologue of *yeaG*, is found in some Gram-positive bacteria such as *Bacillus* or *Clostridium* species. The product of *yeaH*, YeaH, is a 49 kDa uncharacterised protein with very little sequence or structural similarity to any protein described to date. However, intriguingly, the product of *yeaG*, YeaG, is a 75 kDa protein, which contains a carboxyl-terminal domain that shows strong amino acid sequence homology to Hank's type kinase domains [21] and an amino-terminal domain that resembles an ATPase Associated with diverse cellular Activities (AAA+) domain [22]. Indeed, an earlier study by Tagourti et al. [23] had demonstrated that recombinant YeaG had weak kinase activity *in vitro*. Interestingly, the comparison of protein sequences of bacterial kinases available in the public databases indicates that the domain organisation of YeaG is atypical among Hank's type kinases, potentially suggesting that YeaG might be subject to a novel mode of regulation.

A study by Figueira et al. [24] investigated the role of the *yeaGH* operon in the adaptive response to N starvation in *E. coli*. This work revealed that the viability of Δ*yeaG* and Δ*yeaH E. coli* became considerably compromised under sustained N starvation when compared with the wild-type strain. The viability of a Δ*yeaGH E. coli* strain was comparable to that of the respective single mutant strains, implying that YeaG and YeaH functionally interact in the adaptive response to N starvation. Intriguingly, Figueira et al. discovered that the intracellular concentration of σ^38^ was lower in N-starved Δ*yeaG E. coli* than in the wild-type strain. This was primarily due to the transcriptional dysregulation of two toxin–antitoxin gene pairs (*mqsR*/*mqsA* and *dinJ*/*yafQ*) in Δ*yeaG E. coli*, which likely antagonised a regulatory cascade that was responsible for the accumulation of σ^38^. The study concluded that YeaG acts upstream of σ^38^ and that its absence results in a perturbed adaptive transcriptional response to N starvation due to compromised intracellular σ^38^ levels.

In expanding, the knowledge of how *yeaG* contributes to adaptation to sustained nitrogen starvation, a recent study by Switzer et al. [25] used global gene expression analysis to compare the transcriptomes of wild-type and Δ*yeaG E. coli* growing in batch cultures under N replete conditions (N+), and short- (N-starved for 20 min; N−) and long-term (N-starved for 24 h; N-24) N-starved conditions. Surprisingly, the present study uncovered that, despite the intracellular levels of σ^38^ being different in wild-type and Δ*yeaG E. coli* (see above), the σ^38^ regulon was not dysregulated at N+ or N− and only modestly dysregulated at N-24 in mutant bacteria. A possible explanation for this could be that *E. coli* has evolved ‘failsafe’ mechanisms to — at least partly — compensate for the transcription of σ^38^-dependent genes by utilising Eσ^70^ under conditions where Eσ^38^ availability or activity is compromised. The fact that many σ^70^- and σ^38^-dependent promoters are difficult to distinguish at the level of activity (despite moderate differences at sequence level) might be consistent with such a view. Alternatively, the reduction in the intracellular levels of σ^38^ in Δ*yeaG E. coli* may not be sufficiently low to have a detectable impact on transcriptional output from its promoters [26].

**Figure 3.**
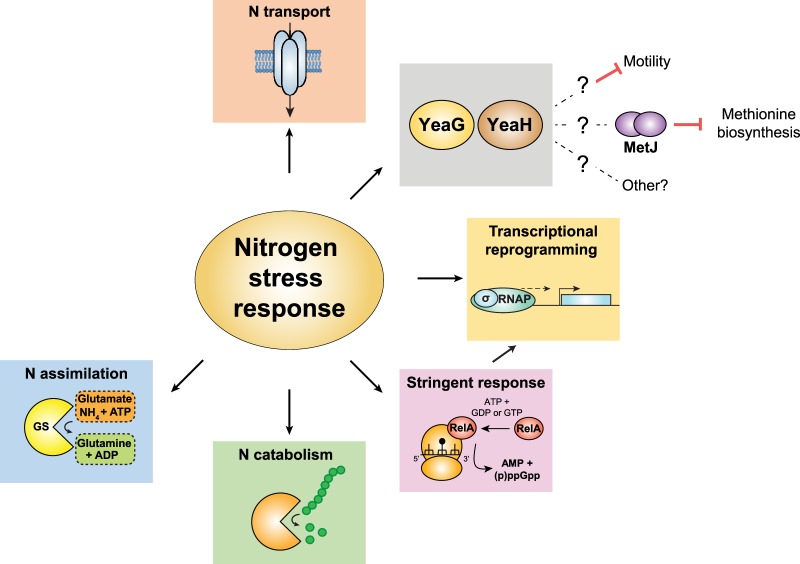
The expanded regulon of NtrC. Summary of the processes affected by the adaptive response to N starvation in *E. coli*, highlighting the role(s) played by the kinase, YeaG.

Intriguingly, Switzer et al. also discovered that the Δ*yeaG E. coli* displayed perturbed phenotypic and metabolic properties, which point to a dysregulation of the adaptive response to N starvation at the transcription level. Firstly, although the adaptive response to N starvation is a scavenging response, it does not involve chemotactic-like behaviour [27]. At N−, shortly after N run-out, several σ^28^-dependent genes, which are associated with the biosynthesis of flagella and motility, become up-regulated in Δ*yeaG E. coli*. Consequently, the mutant bacteria were more motile at N− than their wild-type counterparts. However, at N-24, following a sustained period of time under N starvation, the mutant bacteria ceased being motile. Secondly, the study also uncovered that genes associated with methionine biosynthesis (which are primarily under negative control by the transcription repressor, MetJ) become up-regulated at N-24 in Δ*yeaG E. coli*, consequently resulting in the aberrant biosynthesis of methionine in growth-attenuated and N-starved Δ*yeaG E. coli* cells. Although subsequent analysis revealed that the ability of MetJ to repress transcription (but not to bind to its cognate sites on the DNA) was compromised in mutant bacteria at N-24, the molecular basis by which YeaG affects MetJ activity remains to be elucidated. Since YeaG is a Hank's type kinase, it is tempting to speculate that regulation of MetJ activity during sustained N starvation involves it, or an effector protein, is phosphorylated by YeaG. Similarly, how YeaG influences Eσ^28^ activity at N− warrants further investigation. Clearly, YeaG appears to be a novel regulatory factor affecting the transcriptional regulation of (at least) two distinct genetic networks in N-starved *E. coli*. Thus, we propose that the *yeaGH* operon, alongside *relA*, constitutes a new branch in the Ntr response in *E. coli* (Figure 3).

## Adaptation to N starvation — a dynamic and temporally regulated transcriptional response?

**Figure 4.**
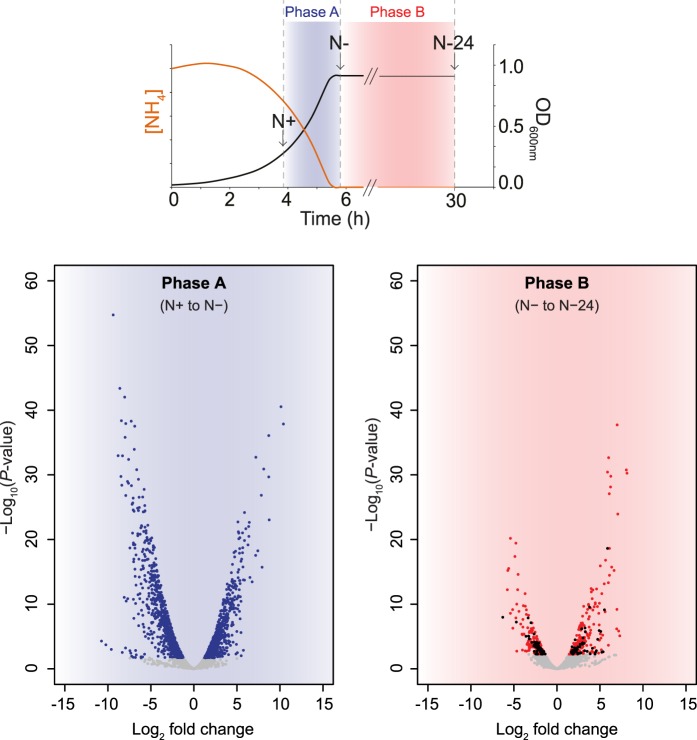
The dynamic adaptive transcriptome of N-starved *E. coli*. Volcano plots illustrating significant differential gene expression shortly (20 min; N−) after N run-out (with respect to N+; phase A; in blue) and following sustained (24 h; N-24) N starvation (with respect to N−; phase B; in red). Significantly differentially expressed genes were defined as those with expression levels changed ≥ twofold with a false discovery rate-adjusted *P*-value < 0.05. The genes that are exclusively differentially expressed in phase B are shown in black.

Perhaps, the most striking observation made by Switzer et al. [25] is that the adaptive transcriptional response in *E. coli* grown in batch cultures is dynamic and subject to temporal regulation (our unpublished observations). For example, upon transition from N+ to N− (phase A), 1787 genes (∼40% of *E. coli* genes) become differentially expressed (Figure 4). However, upon transition from N− to N-24 (phase B), 451 genes (10% of *E. coli* genes) are differentially expressed, of which 105 genes (2% of *E. coli* genes), that were not differentially expressed in phase A, become differentially expressed in phase B (Figure 4). Functional categorisation of the genes specifically differentially expressed in phase B shows that they are involved in diverse processes: transcription, amino acid transport, metabolism and several genes of unknown function. It is therefore tempting to speculate that these 105 genes exclusively contribute to the adaptive response to *sustained* N starvation. It remains to be investigated whether any of the genes that specifically become differentially expressed in phase B are subject to control by NtrC or Nac. The fact that intracellular NtrC levels remain unchanged at N− and N-24 would be consistent with the view that the role of NtrC (and possibly Nac) extends beyond the initial transcriptional response to N starvation. Although the observations made by Switzer et al. do not take the changes in gene expression associated with the massive change in the growth rate following N run-out into consideration, a previous study by Hua et al. [28] studied the transcriptome of N-starved *E. coli* under steady-state conditions (i.e. where the *E. coli* cells are growing at the same specified low growth rate). Their results revealed that only a subset of genes (235) become differentially expressed; just 5% of the *E. coli* genome compared with 40% identified by Switzer et al. that become differentially expressed in response to nitrogen run-out. Most of these genes belonged to the NtrC and Nac regulons. This observation further underscores the need to study the changes the transcriptome of *E. coli* undergoes as N starvation ensues for a longer period of time under steady-state conditions to uncouple changes in gene expression directly associated with adaptation to sustained N starvation from the indirect changes starved, and thus growth-impaired, *E. coli* cells undergo. The study by Switzer et al. ultimately indicates that the adaptive transcriptional response to N starvation occurs in phases to likely allow the cells to both optimally cope with N starvation, and be prepared — at least for a certain period of time — to resume growth when N becomes replenished. The role of YeaG in the adaptive transcriptional response to N starvation, based on the present study, can be considered as ‘molecular brake’, which functions to dampen transcription of ATP-consuming pathways in a temporally co-ordinated manner as the progression of N starvation ensues.

## Perspectives

Although our current understanding of the adaptive response to N starvation in *E. coli* and related bacteria are based on pioneering work from the laboratories of primarily Bob Bender, Martin Buck, Ray Dixon, Sydney Kustu, Boris Magasanik, Mike Merrick, Alexander Ninfa and many others, the recent genome-scale studies point to a complex and temporally co-ordinated and integrated reprogramming of the transcriptome in N-starved *E. coli*. Many details of the newly discovered features of the adaptive transcriptional response to N starvation are still unclear and many more features are likely to be discovered still. Unsurprisingly, the new findings open new questions and provide a springboard for further investigation. In our (perhaps biased) view, we consider the following as priorities:

- YeaG is Hank's type kinase. What are the phosphorylation targets of YeaG in N-starved *E. coli*?
- NtrC and Nac are the master transcriptional regulators of the initial adaptive transcriptional response to N starvation. What is the transcriptional regulatory basis of genes that constitute the transcriptional changes as sustained N starvation ensues?
- Many of the genes of the NtrC and Nac regulons are expressed in pathogenic bacteria during the infection process. To what extent do pathogenic bacteria experience N starvation in the host? To what extent does the adaptive transcriptional response to N starvation differ in the context of the host and that seen in the laboratory setting? How does the dysregulation of the adaptive response to N starvation affect bacterial pathogenesis and infection outcome?
- The transcriptome of N-starved and growth-attenuated bacteria is dynamic. What is the regulatory basis of the temporal changes that the transcriptome undergoes in nutrient-starved growth-attenuated bacteria and what are the phenotypic consequences of its dysregulation?

## Abbreviations

N: Nitrogen
Nac: nitrogen assimilation control
Ntr: nitrogen regulation
RNAP (E): RNA polymerase
